# Supplementary material for: GBCapsNet: A calibrated capsule network for automated gallbladder disease diagnosis via ultrasound imaging
Source: PLoS One. 2026 May 13;21(5):e0348705. doi: 10.1371/journal.pone.0348705 (PMC13170888; doi:10.1371/journal.pone.0348705)
Supplement: Attachment — Submitted filename: Response to Reviewers_sheet_06-04-26.pdf [file pone.0348705.s002.pdf]

Dear Editors,

The authors would like to thank the anonymous reviewers for their constructive comments and suggestions, which significantly improved our manuscript and provided valuable guidance for our research.

We are sharing with you the revised version of the paper. Our response to your comments and our address to them in our paper are enclosed below.

We hope that our revised manuscript is acceptable to you.

Regards & Thanks,

Hareesha K.S (On behalf of other authors)

Responses and changes according to the Academic Editor:

First, thank you so much for your help and support in improving this manuscript.

Reviewer #1 – Comments and Author Responses

We sincerely thank the reviewer for their positive feedback and constructive suggestions. We are pleased that the reviewer finds the manuscript meaningful and improved. We have carefully revised the manuscript to address all the points raised. All modifications have been highlighted in the revised version.

Comment 1: The paper focuses on a meaningful problem and the motivation behind the work is clearly presented.

Response: We thank the reviewer for this encouraging comment.

Comment 2: The proposed approach seems promising, but the explanation of the methodology could be made more simple and clear for better understanding.

Response: Thank you for the notable point. We agree with the reviewer and have revised the **Methodology section** to improve clarity and readability. Technical descriptions have been simplified and better structured to improve understanding.

Comment 3: Some parts of the paper are difficult to follow due to technical complexity, so improving clarity would help readers.

Response: Thank you, sir. We have carefully revised the manuscript to improve overall clarity, including simplifying complex explanations and improving the logical flow across sections (Methodology, Results, and Discussion).

Comment 4: The dataset description is quite limited, more details about data size, sources, and preprocessing would improve reproducibility.

Response: Yes sir,
We have expanded the dataset description to include:

- Total number of images and patients
- Dataset source (UIdataGB)

  (Turki A, Obaid M, Bellaaj H, Ksantini M, AlTaee A. Gallbladder Diseases Dataset. Mendeley Data, V2; 2024. doi:10.17632/r6h24d2d3y.2. source : https://data.mendeley.com/datasets/r6h24d2d3y/2)

- Clarification that the full dataset was used
- Additional details to improve reproducibility

Comment 5: The experimental results are encouraging, but including more performance metrics would make the evaluation stronger.

Response: Thank you for the notable point. We have updated the evaluation discussion by including additional performance-related details and clarifications in the current manuscript. Calibration metrics such as Expected Calibration Error (ECE) have also been clearly presented.

Comment 6: The comparison with existing methods is good, but adding more recent models would strengthen the validation.

Response: Yes, sir, we have updated the comparison section by including more recent models from the literature. At the same time, we have revised the discussion to ensure that comparisons are presented in the current manuscript.

Comment 7: The contribution of each component in the proposed model is not fully clear and could be explained better.

Response: Thank you, sir, for highlighting this critical oversight.
We have clarified the contributions of the proposed model, including:

- The role of capsule networks
- The modified routing mechanism
- The integration of calibration techniques

These have been explicitly described in the revised manuscript.

Comment 8: The paper would benefit from a clear architecture diagram to visually explain the workflow.

Response: Thank you, sir, for this constructive suggestion. A detailed diagram (Fig 2) has been included in the current version of the manuscript.

Comment 9: There are some minor language and grammatical issues that should be corrected to improve readability.

Response: Yes, sir, we have thoroughly revised the manuscript and corrected grammatical errors.

Comment 10: The work is promising overall, and adding discussion on real-time implementation and future scope would further enhance its impact.

Response: Thank you for this suggestion. We have updated the discussion and conclusion sections, which include:

- Future research directions
- Scope for further improvements and validation

Reviewer #2 – Comments and Author Responses

We sincerely thank the reviewer for their detailed and constructive feedback, which has significantly helped to improve this manuscript. We have carefully addressed all comments and revised the manuscript accordingly. All changes have been highlighted in the revised manuscript.

Level 1 (Critical Issues)

Comment 1: 1) Data leakage risk + unrealistic metrics must be addressed experimentally and transparently Image-level splitting is not patient-level splitting and can produce misleading performance. With ~6 images per patient (10K images, 1.7K patients), this will likely have a meaningful effect. This limitation must be clearly stated in the abstract and conclusion, not only in the body text.

The reported performance (99.91% accuracy, AUC 1.0 across 9 classes) is exceptionally high for ultrasound classification, making the leakage/overfitting concern a methodological red flag.

I recommend requesting patient identifiers from dataset creators (if possible) and at least providing full image IDs and split assignments (e.g., in GitHub and/or supplement) to support reproducibility.

Consider an image-level similarity analysis to estimate this leakage, or benchmark on another dataset. At minimum, provide a quantitative discussion estimating the likely degree of leakage and its impact.

Response: Thank you for bringing this important point to our attention regarding potential data leakage. We acknowledge that image-level splitting may introduce intra-patient correlations, potentially leading to optimistic performance estimates.

To address this:

- We have explicitly stated this limitation in the **Abstract, Discussion, and Conclusion**.
- We clarified that the results are based on **image-level splitting**, not patient-level splitting.
- We emphasized that the findings should be considered **preliminary** and require validation using **patient-level splits and independent datasets**.

Comment 2: Unsupported "outperforms existing methods" claims (discussion & conclusion) The manuscript cannot claim the model outperformed existing gallbladder diagnosis methods based on Table 9, which compares accuracy values across different datasets, splits, tasks, and modalities. This is not a controlled comparison and does not support the stated conclusion (and this is central to PLOS ONE expectations). I do appreciate Table 5, which is a valuable contribution.

Response: We appreciate the reviewer for highlighting this critical oversight. We have removed all claims suggesting that the proposed model outperforms existing methods.

- Comparative statements have been revised to be **non-assertive and cautious**.
- A note has been added to Table 9 clarifying that:

"Accuracy values from prior studies are reported on different datasets and evaluation protocols; this table is for illustrative purposes only, not for direct comparison."

- The **Abstract, Discussion, and Conclusion** have been updated accordingly.

Comment 3: Add controlled baselines and uncertainty estimates Add at least one controlled baseline (e.g., ResNet-50 or MobileNet, readily available), fine-tuned on the same dataset, using the same split and same random seed, and report performance alongside GBCapsNet. Add confidence intervals by running multiple splits/seeds and reporting variability of outcomes

Response: We appreciate this valuable recommendation and have incorporated it into the revised manuscript. Due to computational and time constraints, we were unable to include additional controlled baseline experiments (e.g., ResNet-50 or MobileNet) or perform multiple runs for uncertainty estimation in this revision.

However:

- We have **explicitly acknowledged this limitation** in the manuscript.
- We have clearly stated that **no controlled baseline comparisons were conducted**, and this limits the strength of comparative conclusions.
- We have identified this as an important direction for **future work**.

Comment 4: Reproducibility and PLOS ONE code/data availability expectations While the dataset is named, the GitHub is not referenced within the manuscript, and the current setup is not sufficient for full reproducibility. PLOS ONE requires code availability stated in the manuscript. Add a clear statement and include explicit train/test split assignments (requested in round 1). Ideally provide this, and code, as supplementary material so it is linked to the DOI. Clarify whether the full dataset was used or only a subset.

Response:
We have added a dedicated section titled **"Data and Code Availability"** in the manuscript.

This includes:

- Dataset details (UIdataGB, 10,692 images, 1,782 patients) https://data.mendeley.com/datasets/r6h24d2d3y/2
- Confirmation that the **entire dataset was used**
- Public GitHub repository link containing (https://github.com/madhu76/GBCapsNet-AI)
    - Training and evaluation code
    - Train/test split scripts
    - Model configuration details

This ensures full reproducibility in accordance with journal requirements.

Comment 5: Calibration protocol clarity and structure
Clarify: what fraction of training data is held out for calibration, and how was it selected?
Why is calibration not shown for the primary 90–10 split?
Check abstract statements against Table 8 (they appear contradictory).
Calibration methods belong in Methods, calibration outcomes in Results (currently there is overlap).

Response: Thank you for bringing this important point to our attention.
We have revised and clarified the calibration methodology:

- Moved calibration details to the **Methods section**
- Clearly specified:
    - Use of **temperature scaling**
    - Validation subset used for calibration
    - Applicable train-test splits
- Clarified that calibration was not applied to the 90–10 split due to insufficient test size

The Abstract and Results have been updated for consistency.

Level 2 (Important)

Comment 6: Abstract/conclusion must reflect limitations and experimental scope If leakage/baseline experiments cannot be run, this must be reflected explicitly in the discussion, abstract, and conclusion. In the conclusion: separate (i) concise summary of results, (ii) honest interpretation/limitations, and (iii) future work.

Response: Thank you for the notable point. We have revised both the Abstract and Conclusion to explicitly reflect: Data leakage risk due to image-level splitting, Absence of controlled baselines, and Preliminary nature of findings. The tone has been made more cautious and scientifically balanced.

Comment 7: Clinical/implementation section is too long and generic

"Design considerations for clinical use" spans 2 pages but is largely generic (POCUS, DICOM, PACS, OSI networking). Condense to one paragraph or at most two in the discussion to improve focus and flow.

Response: Thank you for the notable point. We have revised this section and removed/commented out non-essential content based on reviewer feedback from previous rounds. To avoid confusion, all such commented content has now been **fully removed from the manuscript**.

Comment 8: Ethics statement

Even if formal ethics approval is not required, briefly explain the reasoning in the manuscript for stronger transparency, it could for example be below the data statement.

Response: Yes sir. We have added a dedicated Ethics Statement: This study used an open-source, fully anonymized gallbladder ultrasound dataset. As no identifiable patient information was used, formal ethics approval was not required.

Comment 9: Optional but helpful: additional reporting Consider supporting Table 6 with a confusion matrix. Explicitly state whether data augmentation was used.

Response: We thank the reviewer for the suggestion. Updated and confirmed that **data augmentation was not used** in this study and have clarified this in the manuscript. A confusion matrix is not included in the current version; we acknowledge this limitation and will consider including it in future work.

Level 3 (Minor editorial corrections)

Comment 10: Terminology and factual consistency

"Ultrasound is minimally invasive" is not generally correct; it depends on modality (e.g., EUS can be minimally invasive). Decide what applies here (EUBUS? transabdominal?) and use consistent terminology.

OSI model: clarify whether you mean 5 layers vs 7 layers (currently confusing).

Response: Thank you for this notable point. We have clarified that our study focuses on transabdominal ultrasound imaging, which is a non-invasive diagnostic modality. We have removed the term ‘minimally invasive’ throughout the manuscript. Verified that no incorrect references (e.g., OSI model confusion) or information remain in the manuscript. Removed residual commented text that could cause confusion

Comment 11: Abstract and language corrections
“the findings suggest” → “the findings suggest” (plural agreement: findingS suggest).
“capsule based” → “capsule-based”
“Cholestrol” → “cholesterol”
CNN layers: they are typically convolutional, not “fully connected” as stated (unless you refer to a specific head; please clarify).
Author contributions: remove extra comma in “Chandrika, , A. Sai…”
“Related work” is a better header than “previous works”.

Response: Thank you, sir. We appreciate the reviewer’s point regarding the description of CNN layers. In the revised manuscript, we have clarified that the initial feature extraction stages consist of convolutional layers. We have restricted the term 'fully connected' strictly to the dense layers following the DigitCaps layer, ensuring the architectural description aligns with standard deep learning terminology.

Comment 12: References and citation quality
Reference 19 (flagged in round 1) is still a Medium blog post; not acceptable as a core reference. Please replace with peer-reviewed works.
Reference 2 is a preprint: acceptable if necessary, but label it as a preprint in references.
Reference metadata issues: Ref 20 is NeurIPS 2017 (not preprint); similarly verify Ref 16 (MICCAI 2015), 17 (NeurIPS 2015), 46 (ICML 2017). Please double-check all references.

Response: We thank the reviewer for pointing out the inappropriate use of a blog post as a core reference. We have updated the current manuscript.

Comment 13: Formatting/consistency
“Lecun normal” → “LeCun …”
Figs 6 and 7 have identical captions; please differentiate them.

Response: We appreciate the reviewer for identifying the identical captions for Figures 6 and 7. This is updated in the current manuscript.

*****
