# Supplementary material for: GBCapsNet: A calibrated capsule network for automated gallbladder disease diagnosis via ultrasound imaging
Source: PLoS One. 2026 May 13;21(5):e0348705. doi: 10.1371/journal.pone.0348705 (PMC13170888; doi:10.1371/journal.pone.0348705)
Supplement: Attachment — Submitted filename: Response to Reviewers_sheet_03-03-26.docx [file pone.0348705.s001.docx]

Dear Editors,

The authors would like to thank the anonymous reviewers for their constructive comments and suggestions, which significantly improved our manuscript and provided valuable guidance for our research.

We are sharing with you the revised version of the paper. Our response to your comments and our address to them in our paper are enclosed below.

We hope that our revised manuscript is acceptable to you.

Regards & Thanks,

**Hareesha K.S** (On behalf of other authors)

**Responses and changes according to the Academic Editor:**

First, thank you so much for your help and support in improving this manuscript.

Q.1. Please note that PLOS is unable to publish screenshots of commercial websites or applications under a CC BY 4.0 license. Before we can proceed, we ask that you please remove any screenshots of commercial websites/applications in Figures 6 and 7. If you would like to include these images in a non-screenshotted format, please resubmit them as images that are not screenshotted and do not include any imagery from a commercial website/application.

To view PLOS One's policies regarding licenses and copyright, please use this link: <https://journals.plos.org/plosone/s/licenses-and-copyright>

**Response:**

Thank you for your detailed guidance regarding Figures 6 and 7

Removed figures 6 and 7 due to copyright issues, which are updated in the current version of the manuscript.
